# Neurally adjusted ventilatory assist reduces asynchrony and patient effort in severe acute respiratory distress syndrome patients undergoing extracorporeal membrane oxygenation

**DOI:** 10.1186/cc9611

**Published:** 2011-03-11

**Authors:** T Mauri, G Bellani, A Confalonieri, F Magni, G Grasselli, N Patroniti, A Pesenti

**Affiliations:** 1 Universita degli Studi di Milano-Bicocca, Monza, Italy; 2 San Gerardo Hospital, Monza, Italy

## Introduction

Assisted ventilation may prevent muscle atrophy and reduce sedation needs in severe acute respiratory distress syndrome (ARDS) patients undergoing extracorporeal membrane oxygenation (ECMO). However, pressure support (PS) is difficult to implement in these patients: inspiratory flow peaks and drops rapidly and the ventilator expiratory phase may overlap patient inspiration causing asynchrony and barotrauma. Neurally adjusted ventilatory assist (NAVA) is an assisted ventilation mode driven by diaphragmatic electrical activity (EAdi) and should adapt better to patients' respiratory pattern. We measured whether NAVA could reduce asynchrony in severe ARDS patients undergoing ECMO.

## Methods

We enrolled seven consecutive adult patients undergoing ECMO for severe ARDS. Twenty-four hours after their ventilation mode was switched from controlled to assisted, we randomly tested the following strategies for 30 minutes each, leaving positive end-expiratory pressure (PEEP), FiO_2 _and ECMO settings unchanged: (1) PS with expiratory trigger at 30% of flow peak value (PS30); (2) PS with expiratory trigger at 1% (PS1); (3) NAVA. The PS level and NAVA gain were chosen to obtain a similar tidal volume (V_T_). From continuous recordings of airway pressure, flow, volumes and EAdi we calculated the average V_T, _respiratory rate (RR) and asynchrony index (AI: number of asynchrony events/(ventilator cycles + wasted efforts) × 100) of each step and, at the end, we measured arterial blood gases and p0.1. Data are the median (IQR) and were compared by nonparametric Friedman test and linear regression.

## Results

At enrolment, patients were 44 (42 to 56) years old. Respiratory system compliance (Crs) was 12 (9 to 23) ml/cmH_2_O, PEEP 10 (7 to 12) cmH_2_O, FiO_2 _0.5 (0.4 to 0.5) and V_T _2.9 (2.8 to 4) ml/kg. Patients were on 3.2 (2.9 to 3.6) l/minute venovenous ECMO since 22 (16 to 29) days. Switching from PS30 to PS1 to NAVA, PaO_2_/FiO_2 _did not change (*P *= 0.817), p0.1 was reduced (3.1 (2.6 to 5.7) vs. 2.1 (1.8 to 3.2) vs. 1.6 (0.9 to 2.3) cmH_2_O, *P *= 0.003) together with RR (*P *= 0.129) and AI (55 (29 to 66) vs. 46 (26 to 56) vs. 16 (8 to 18)%, *P *= 0.004). The difference between AI during PS30 and NAVA was significantly correlated with Crs (*R*^2 ^= 0.87, *P *= 0.02).

## Conclusions

Implementation of NAVA in severe ARDS patients undergoing ECMO may decrease patient effort and asynchrony events. The advantage of NAVA over PS is more evident in patients with lower Crs.

